# A Real Pioneer

**Published:** 1920-11-06

**Authors:** 


### THE MEDICAL EDUCATION OF WOMEN.

### A Real Pioneer.

It is an ungrateful, but seems a very necessary task, to point out who was really the pioneer in the medical education of women. Dr. Mary Garrett Anderson and Dr. Sophia Jex Blake were certainly pioneer women doctors, and at the luncheon on Tuesday, October 19, to commemorate the near approach of the jubilee of the London (Royal Free Hospital) School of Medicine for Women (University of London), it was right that their names should have been mentioned. But it was not right that the name of the pioneer lady, Dr. Elizabeth Blackwell, should have been omitted on such an occasion. Her most interesting autobiography, "Pioneer Work for Women," by Dr. Elizabeth Blackwell, with supplementary chapter, and introduction by Mrs. Fawcett, is available in a new edition in Dent's Everyman Library. Therein it is related how she conceived and planned her scheme of becoming a doctor of medicine and gaining a place on the Medical Register when she was absolutely penniless and her family in the same position. How she did it, British-born as she was, in an American University is one of the heroic stories of the nineteenth century. Her vigorous and well-informed "Essays in Medical Sociology," which might still be the reference book of the social reformer, were published by Ernest Bell, London, in 1902. Her dust rests in Kilmun Churchyard, Argyllshire, behind the mausoleum of Douglas of Glenfinnart, and not far from that of the Argyll family. The spot is marked by a handsome marble Celtic cross bearing this inscription:

"In loving memory of Elizabeth Blackwell, M.D., born at Bristol, 3rd February, 1821, died at Hastings, 31st May 1910.

The first woman of modern times to graduate in medicine (1849) and the first to be placed on the British Medical Register (1859)."

Here follow two Scripture texts and a quotation from her lecture on "The Religion of Health." "It is only when we have learned to recognise that God's law for the human body is as sacred—nay, is one with—God's law for the human soul that we shall begin to understand the religion of the heart." Hobart College, Geneva, New York, where she graduated, named its first dormitory for women after her. In twenty years from the time of her graduation medical schools for women were established in Boston, Philadelphia, and New York. When she saw New York Infirmary and Medical School well established these were left in charge of her sister, Dr. Emily Blackwell, and a corps of capable professors, and she crossed to Great Britain to inaugurate and inspire a like work there. The careers of Dr. Garrett Anderson and Dr. Jex Blake were some of the first fruits of her labours and of those lectures she delivered in Marylebone in 1859. The New Hospital and London School of Medicine for Women had its origin in the medical dispensary established in Seymour Place by Miss Garrett afterwards Dr. Garrett Anderson. For a time Dr. Blackwell held the chair of gynæcology here. Her autobiography, "Pioneer Work," was first issued in 1895. One who read it at the time wrote: "If the young people would only put a little of the moral qualities into their work that you put into yours when you set out on the lonely way which was to become, thanks to you, a beaten path, how glorious women's work would soon become, and how helpful to poor humanity."

The name of Elizabeth Blackwell was placed on the British Medical Register before Dr. Garrett Anderson began to study medicine, and yet in all the reports of the jubilee of the New Hospital for Women this lady is called the first English woman doctor of modern times. Miss Katherine Barry, adopted daughter of Dr. Elizabeth Blackwell, handed over her diploma of 1849 to the custody of Queen Margaret College, Glasgow, in 1913.

